# A case of intraductal papillary neoplasm of the bile duct arising in Lynch syndrome resected under an endoscope

**DOI:** 10.1055/a-2599-9783

**Published:** 2025-06-03

**Authors:** Shanshan Shen, Shuang Nie, Lei Wang

**Affiliations:** 1 66506 Department of Gastroenterology, Nanjing Drum Tower Hospital, Affiliated Hospital of Medical School, Nanjing University, Nanjing, China

A 65-year-old female found a hilar bile duct mass with associated biliary dilation under CT and MRI during routine surveillance. Notably, the patient had a complex medical history, receiving segmental liver resection 8 years ago for intraductal papillary neoplasm of the bile duct (IPNB), and right hemicolectomy 7 years ago for mucinous adenocarcinoma. Additionally, the patient had a strong family history, including cardia cancer in her father and stomach cancer in her brother.

At the recent visit, endoscopic ultrasound (EUS) identified a lobulated, hyperechoic lesion (16.5 mm × 17.2 mm) at the remnant common bile duct ( Fig. 1 ), subsequently confirmed under endoscopic retrograde cholangiography (ERC) and cholangioscopy ( Fig. 2 ). Given the patient’s history of two major surgeries, she was reluctant to repeat surgical intervention and opted for an endoscopic approach. Considering the absence of malignant features and significant bile duct dilation (18 mm), a staged EUS-guided approach was feasible. First, EUS-guided cholangioduodenostomy (EUS-CDS) was performed using a 15 mm × 10 mm lumen-apposing metal stent to establish a duodeno-biliary fistula. Two weeks later, the stent was removed, and the tumor was resected via endoscopic mucosal resection (EMR) through the fistula ( Fig. 3 ), followed by argon plasma coagulation (APC) ablation for residual lesions. The patient showed no recurrence under cholangioscopy at the 6-month follow-up ( Fig. 4 , Video 1 ).

**Fig. 1:**
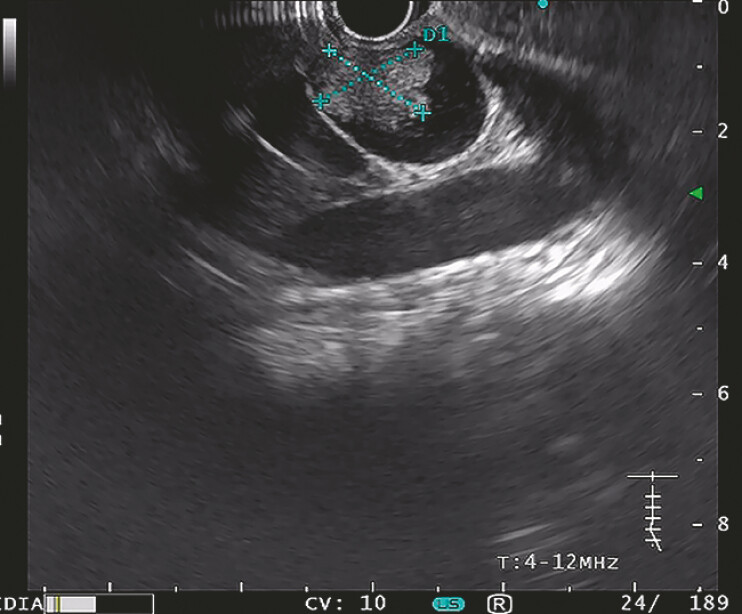
EUS identified a lobulated, hyperechoic lesion (16.5 mm × 17.2 mm) at the remnant common bile duct. Abbreviation: EUS, endoscopic ultrasound.

**Fig. 2:**
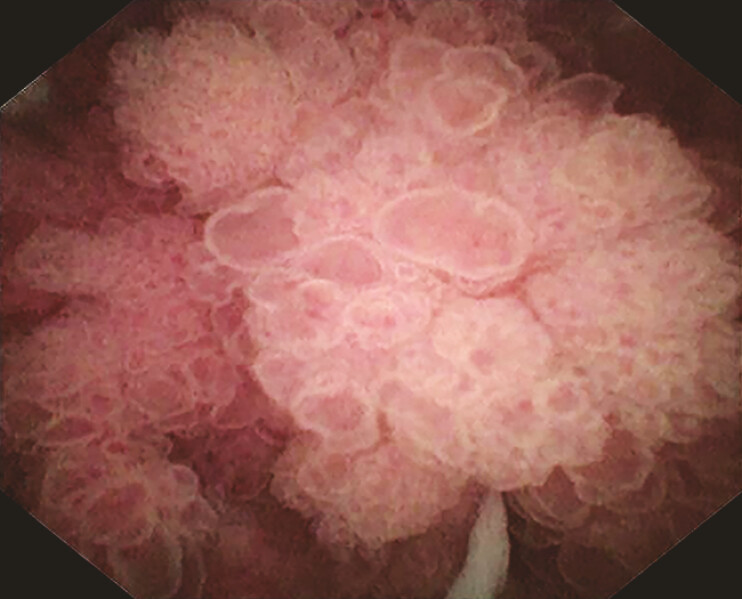
ERC and cholangioscopy confirmed the remnant common bile duct mass. Abbreviation: ERC, endoscopic retrograde cholangiography.

**Fig. 3:**
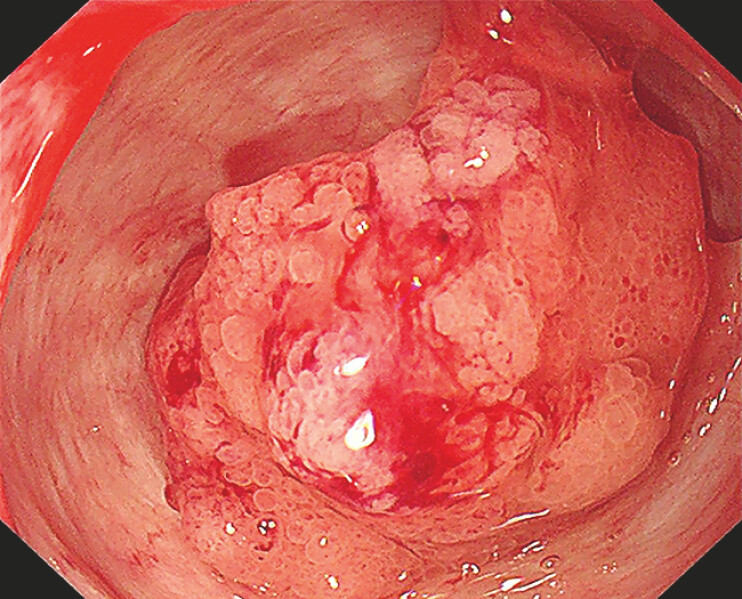
The tumor was visualized via endoscopy through the fistula.

**Fig. 4:**
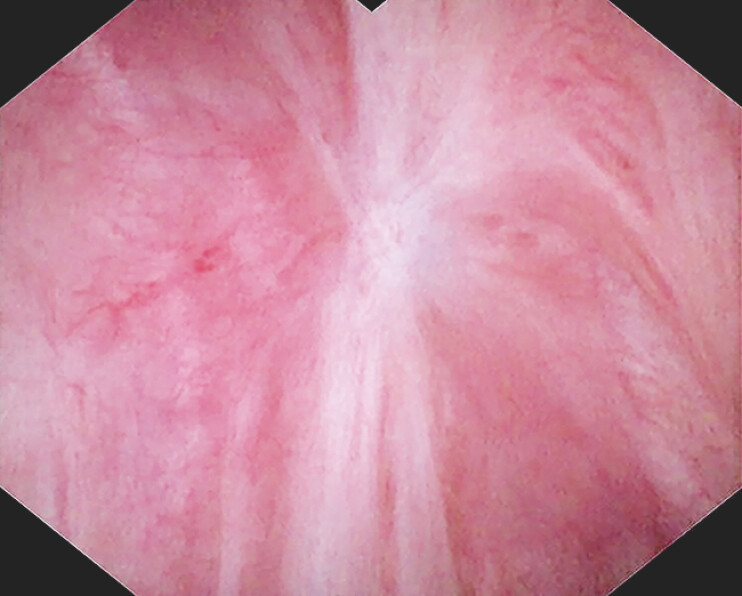
The scar showed no recurrence after EMR and APC after 6 months. Abbreviation: APC, argon plasma coagulation; EMR, endoscopic mucosal resection.

A case of intraductal papillary neoplasm of the bile duct arising in Lynch syndrome resected under an endoscope.Video 1

Given her personal medical and family history, Lynch syndrome was suspected. Pathology acquired under endoscopy confirmed IPNB with focal high-grade intraepithelial neoplasia. Furthermore, immunohistochemistry (IHC) together with comprehensive genomic profiling identified a germline pathogenic MSH2 variant, confirming the diagnosis of Lynch syndrome 1 ( Fig. 5 ).

**Fig. 5:**
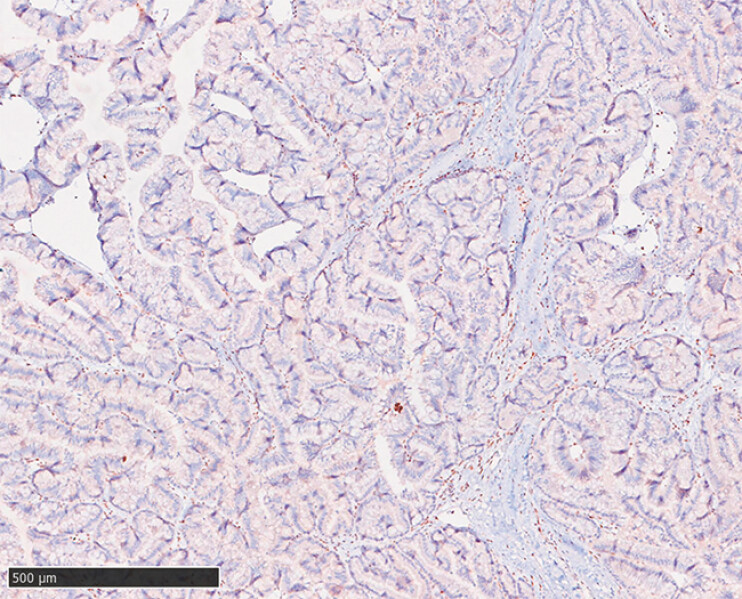
IHC showed the absence of MSH2 protein. Scale bar: 500 µm. Abbreviation: IHC, immunohistochemistry.

This case highlights the first complete endoscopic resection of IPNB, with early tumorigenesis arising in Lynch syndrome. It successfully provides new ideas for the early diagnosis and treatment of this complex condition.

Endoscopy_UCTN_Code_TTT_1AR_2AF

## Notes

Endoscopy E-Videos https://eref.thieme.de/e-videos
*E-Videos* is an open access online section of the journal *Endoscopy* , reporting on interesting cases and new techniques in gastroenterological endoscopy. All papers include a high-quality video and are published with a Creative Commons CC-BY license. Endoscopy E-Videos qualify for HINARI discounts and waivers and eligibility is automatically checked during the submission process. We grant 100% waivers to articles whose corresponding authors are based in Group A countries and 50% waivers to those who are based in Group B countries as classified by Research4Life (see: https://www.research4life.org/access/eligibility/ ). This section has its own submission website at https://mc.manuscriptcentral.com/e-videos .
